# Spatial Correlation Network and Regional Differences for the Development of Digital Economy in China

**DOI:** 10.3390/e23121575

**Published:** 2021-11-25

**Authors:** Luyang Tang, Bangke Lu, Tianhai Tian

**Affiliations:** 1 School of Business Administration, Zhongnan University of Economics and Law, Wuhan 430073, China; tangluyang@stu.zuel.edu.cn; 2 School of Statistics and Mathematics, Zhongnan University of Economics and Law, Wuhan 430073, China; 3 School of Mathematics, Monash University, Melbourne, VIC 3800, Australia; tianhai.tian@monash.edu

**Keywords:** digital economy, regional differences, north and south, network analysis, correlation effect

## Abstract

The rapid development of the digital economy is a powerful driving force to promote high-quality economic growth all over the world. Although a number of studies have been conducted to investigate the development of the digital economy in China, these studies pay little attention to the spatial linkages between the 30 provinces in China and the developmental differences between northern and southern China. Using Chinese digital economic data from 2004 to 2019, we propose an index system to measure the developmental levels of the digital economy and obtain the annual developmental levels of these provinces by using the factor analysis method. We analyze the regional differences of developmental levels by using the Theil index and kernel density estimation method. More importantly, the network method is used to analyze the correlations between the developmental levels of the digital economy in all provinces of China. By decomposing regional differences, our study shows that polarized and uncoordinated development is prominent. The development level of the digital economy in the southern region is higher than that in the northern region. In terms of regional correlations, the network study suggests that there are beneficial and spillover effects of the digital economy development between provinces. Based on the analysis results, we propose policies for improving the development of the digital economy in China.

## 1. Introduction

In recent years, network science has become an important method to study the internal relationship and regulation mechanism of complex economic systems. The premise of understanding the mechanism of economic development is to measure the structural complexity of the entire system, which is made easier by constructing complex networks based on economic physics [1,2]. Complex network refers to the existence of many nodes or variables in the system and the relationship between these nodes [3]. Digital economy is an advanced stage of the development of information economy and informatization, and the flow of information is complex and uncertain. The use of complex network analysis can effectively explain the internal connections of China’s digital economy development system.

Digital economy refers to the economy that uses digital computing technologies. The term ‘digital economy’ was first mentioned in Japan and coined in Don Tapscott’s 1995 book [4]. Although it is normally assumed that the digital economy conducts economic activities using the internet and the World Wide Web, it is intertwined with the traditional economy, making a clear delineation harder.

The improvement of digital skills can effectively promote economic growth [5,6,7]. However, the outbreak and spread of COVID-19 have increased the uncertainty of the global economy substantially. In this context, the competition of comprehensive national strength regarding the digital economy is becoming increasingly fierce. At present, both developed and developing countries focus on digital governance, taking the acceleration of the digital economy as a major strategy for economic and social development. In the digital era, the revolution of digital technologies is making a global digital division. However, developing countries can still make full use of the digital economy to narrow the gap between themselves and developed countries [8,9,10].

In recent years, the digital economy has rapidly developed in China. It has been an important power to stimulate the growth of the Chinese economy. According to the white paper on the global digital economy, issued by the China Institute of Information and Communication in 2020, the scale of the digital economy in the United States far exceeded the global level and continued to rank first in the world. In addition, China ranked second in the world with $5.4 trillion US dollars with a year-on-year growth rate of 9.6%, ranking first in the world regarding growth rate. There is still a gap between China and the United States regarding the digital economy, which is mainly reflected by digital infrastructure and talent innovation [11].

Although the digital economy has been developed well in China, the domestic gap is still significant [12]. The difference is presented not only in urban and rural areas, but also in different regions in China. For example, digital enterprises are mainly located in the major cities in the eastern and middle regions of China. It is expected that sustained high growth of the digital economy will further widen the economic gap between different regions [13]. The expanding imbalance may lead to not only economic challenges but also serious social problems. In addition, undeveloped areas may further hinder the overall development of the national economy. In fact, the regional differences in digital economy development are also a substantial challenge to the world economy. Some research studies have investigated the impact of the unbalanced development of the digital economy on the growth of the regional economy in different countries [14,15,16]. In China, the economic development gap between the northern and southern parts has been widened in recent years [17]. The question is whether the digital economy has a north-south gap which will be the engine of future economic development. Therefore, it is urgent to study the regional differences of digital economy development between northern and southern China, analyze the reasons of such regional differences, and propose policies to promote balanced development.

There are two major issues in studying regional differences of digital economy development. The first one is how to measure the developmental level of the digital economy. There are a number of problems in measuring the digital economy, including the distorted boundaries between the digital sector and other economic sectors, poor data quality, pricing issues, and the unobservable nature of digital activities [18]. At present, there are few studies about the comprehensive evaluation and analysis of the development levels. The published evaluation studies dominantly focus on the theoretical issues, and few applicable systems have been applied for economic data. To address this issue, this work will design an evaluation index system to assess the digital economy developmental level by the factor analysis method using the data of 30 provinces in China from 2004 to 2019. In particular, we propose to use digital innovation capability as a key index, which is the driving force for the development of digital economy.

The second issue is how to quantitatively evaluate the regional differences and how to analyze the spatial correlation between provinces. Related questions include how the northern and southern regions are divided, what methods are used to measure regional differences, and whether there are links between the regional or provincial development of the digital economy. Taking the Qinling-Huaihe line as the north-south boundary, this paper divides 30 provinces into northern and southern regions. We first use the Theil index and kernel density method to analyze the regional difference of the digital economy development level. The innovations of this study also include the application of network analysis to explore the spatial relationships of digital economy development between different provinces and discuss both the benefit and spillover correlation effects.

Subsequent aspects of this paper are organized as follows. Section 2 provides a literature review. Section 3 discusses the economic data, data processing methods, and empirical models in detail. Section 4 presents the empirical results of the regional differences and spatial correlation network in the digital economy development in China. Conclusion and discussions are provided in Section 5.

## 2. Literature Review

Digital economy includes two forms of economic transformation, namely digital industrialization and industrial digitalization. As an emerging economic and social form, the digital economy has surpassed the boundaries of information industry and internet technology. Principal digital economy combines technology, industry, producers, and consumers to form an innovative economic growth model [19,20,21,22]. In the Digital Economy Outlook 2017, the Organization for Economic Cooperation and Development (OECD) defines digital economy as the digital transformation of economic and social development from a strategic perspective of digital economy development [23].

Digital economic accounting is fundamental for measuring digital economy. Kang developed the general structure of digital economic accounting that should include the investment data, network transaction data, and business data of enterprises in the digital economy [24]. Based on the digital economy in Cameroon, Etoundi et al. discussed the important indicators related to the development of the digital economy, such as civil society, services and commodities, policies, regulations, and basic technological infrastructure [25]. Cai proposed an improved method combining growth accounting and conventional GDP accounting for the scale accounting of digital economy. This method first calculates the total amount of digital economy based on the increase in the digital economy and then measures the developmental scale of digital economy based on the contributions of digital economy to GDP [26]. Wen et al. quantified the development level of digital economy using the number of telephone users (both fixed and mobile phones), the number of broadband users and telecommunication service income [27]. Shan et al. proposed a three-dimensional evaluation system included information cyberspace, physical space, and human social space to construct a comprehensive index system for the developmental level of digital economy [28].

For a long time, China’s economy has been divided into four regions, namely the east, middle, west, and northeast regions. This is because the economic development stages, growth models, and development policies of these four regions are different. However, in recent years, the economic development among the four regions has not shown much contrast. On the contrary, the economic difference between the north and south is widening. The difference in the regional economic development changes from the east-west difference to north-south difference [29]. The most important factor lies in the difference in the regional innovation ability [30]. The question is whether the innovation gap between the south and north leads to the difference in digital economy development between the two regions. To answer this question, Yang & Jiang [31] used the principal component analysis and factor analysis to measure the development level of the digital economy in 30 provinces and analyzed the regional differences between the north and south.

Research studies and economic data show that the development level of China’s digital economy has been increased each year. According to the classification of economic belts, there is significant heterogeneity among the Beijing-Tianjin-Hebei coordinated development economic belt, the Yangtze River economic belt, the ‘Belt and Road’ construction economic belt, the Yangtze River delta integration economic belt, and the yellow river basin economic belt. According to the regional division, it is found that there are obvious regional differences in the development of the digital economy in the eastern, central, western, and northeastern regions [32,33]. However, few scholars pay attention to the north-south difference in the development of China’s digital economy. The motivation of this study is to fill this research gap.

In fact, the digital economy in each region or each province is in dynamic development. In addition to regional differences, there are also certain economic relationships between different regions [34]. Therefore, this paper uses the network analysis method to study the spatial correlation of China’s digital economy development.

## 3. Data and Methods

### 3.1. Index System of Digital Economy

Yang & Jiang [31] recently designed an index system for the development level of the digital economy from the perspective of industrial digitalization and digital industrialization. This system neglects the factors related to research and development, which is the driving force of the digital economy. We first propose a comprehensive index system that includes not only the innovation of digital technology but also a broader range of digital industry. The proposed index system measures the development level of the digital economy from four aspects: digital infrastructure, digital innovation ability, digital industry scale, and digital technology application.

We select 17 s-level indexes, which are given in Table 1. Digital infrastructure based on information and communication technology is a prerequisite for the development of the digital economy. The quality of digital infrastructure has a direct impact on the development of digital economy. Organizations and scholars all choose digital infrastructure as a key index when measuring the digital economy. Under this first-level index, there are four second-level indicators. The second first-level index is the digital innovation capability that measures the development potential to support the development of this very competitive economic sector. There are three second-level indicators under this first-level index. The scale of digital industry, as the third first-level index, is a clear indicator of the output level of digital economy and directly reflects the development level of digital economy. There are five second-level indicators under this first-level index. Finally, we choose the application of digital technologies as the fourth first-level index. The power of digital economy is to change the business model and benefit people’s daily life via convenient digital services. Table 1 summarizes the information of the first-level and second-level indicators.

### 3.2. Digital Economy Data

We collect the data from the China Statistical Yearbook, China Information Industry Yearbook, China Information Yearbook, China Science and Technology Statistical Yearbook, Statistical Yearbook of Provinces and Provinces, and the website of the National Bureau of Statistics. Some missing data are estimated by interpolation. This work analyzes the digital economy activities of 30 provinces (excluding Tibet due to the incomplete data) from 2004 to 2019. For simplicity, municipality and autonomous region all are referred as province in this study. Taking Qinling-Huaihe line as the north-south boundary, these 30 provinces are divided into two regions, which is given in Table 2. As an example, Table A1 in the Appendix A gives the data of Beijing for the 17 s-level indicators from 2013 to 2018.

### 3.3. Index of Indicators

In this paper, the factor analysis method is used to measure the development level of each province. This method synthesizes a few comprehensive common factors by studying the correlation between a group of indicators and uses these common factors to represent a linear model of original variables.

Based on the index system in Table 1, this paper conducts a factor analysis to evaluate the four first-level indicators. The factor analysis is applied to the second-level indicators corresponding to each first-level indicator. Then we obtain the evaluation score of each first-level indicator. The dataset includes the information of four indexes for 30 provinces over 16 years. Here $x_{ij}\;\left(i=1,2,\cdots 30;\;j=1,2,3,4\right)$ denotes the raw data that make up the samples, and $x_{.j}$ is the observed value of the j-th index. We suppose that each index has k common factors $F_{1},F_{2},\cdots ,F_{k}$. The influences that cannot be explained by the factor are expressed as $\varepsilon_{1},\varepsilon_{2},\varepsilon_{3},\varepsilon_{4},\;\mathrm{respectively}.$ The factor analysis model is expressed as.

(1) $$x_{.1}=a_{11}F_{1}+a_{12}F_{2}+\cdots a_{1k}F_{k}+\varepsilon_{1}\;x_{.2}=a_{21}F_{1}+a_{22}F_{2}+\cdots a_{2k}F_{k}+\varepsilon_{2}\;x_{.3}=a_{31}F_{1}+a_{32}F_{2}+\cdots a_{3k}F_{k}+\varepsilon_{3}\;x_{.4}=a_{41}F_{1}+a_{42}F_{2}+\cdots a_{4k}F_{k}+\varepsilon_{4}$$

with component matrix $A=\left[\begin{array}{ccc} a_{11} & \cdots & a_{1k}\\ \vdots & \vdots & \vdots \\ a_{41} & \cdots & a_{4k} \end{array}\right]$ and $\varepsilon ={\left(\varepsilon_{1},\varepsilon_{2},\varepsilon_{3},\varepsilon_{4}\right)}^{\prime }$. Therefore, the factor analysis model is represented by X=AF+ε. The common factor $F_{k}\;$is given by

(2) $$F_{k}=\overset{4}{\underset{j=1}{\sum }}c_{hj}x_{.j}\left(h=1,2,\;\cdots k;j=1,2,3,4\right)$$

where $c_{hj}$ is the unknown coefficients of common factor $F_{k.}$ The comprehensive score of digital economy development in i-th province is given by

(3) $$Y_{i}=\overset{4}{\underset{j=1}{\sum }}w_{j}F_{ij}\left(h=1,2,\;\cdots k;j=1,2,3,4\right)\;$$

where the weight $w_{j}$ is the variance contribution rate of the j-th factor divided by the cumulative variance contribution rate.

### 3.4. Theil Index and Subgroup Decomposition

Theil (1967) proposed the Theil index (also known as Theil entropy) to study the income gap between countries [35]. The larger the Theil index, the greater the regional differences. Regional differences can be studied by changing countries into regions, and the Theil index can decompose the total differences between regions into inter-regional differences and intra-regional differences. With N(=30) provinces, the formula for the total Theil index in year *t* is given by

(4) $$T_{t}=\overset{N}{\underset{i=1}{\sum }}\left(\frac{D_{it}}{D_{t}}\right)ln(\frac{D_{it}/D_{t}}{P_{it}/P_{t}})$$

where $D_{it}\left(D_{t}\right)$ is the digital economy development level of province i (the whole nation) in year t, $D_{t}=\sum_{i=1}^{N}D_{it}$, $P_{it}\left(P_{t}\right)$ is the number of permanent residents of province i (the whole nation) in year t, and $P_{t}=\sum_{i=1}^{N}P_{it}$.

The southern (northern) Theil index in year *t* is given as follows:

(5) $$T_{st}=\overset{N_{s}}{\underset{i=1}{\sum }}\left(\frac{D_{it}}{D_{st}}\right)ln(\frac{D_{it}/D_{st}}{P_{it}/P_{st}})$$

(6) $$T_{nt}=\overset{N_{n}}{\underset{i=1}{\sum }}\left(\frac{D_{it}}{D_{nt}}\right)ln(\frac{D_{it}/D_{nt}}{P_{it}/P_{nt}})$$

where $D_{st}\left(D_{nt}\right)$ is the digital economy development level of South China (North China) in year t, $D_{st}=\sum_{i=1}^{N_{s}}D_{it}$, $D_{nt}=\sum_{i=1}^{N_{n}}D_{it}$, $N_{s}=15,N_{n}=15.$ $P_{st}\left(P_{nt}\right)$ is the number of permanent residents of South China (North China) in year t, $P_{st}=\sum_{i=1}^{N_{s}}P_{it}$, $P_{nt}=\sum_{i=1}^{N_{n}}P_{it}.$

The decomposition method divides the Theil index $T_{t}$ into the intra-regional difference $T_{wt}$ and inter-regional difference $T_{bt}$, namely $T_{t}=T_{wt}+T_{bt}$. In this paper, the intra-regional difference contribution $T_{wt}$ represents the difference of developmental levels among provinces in the same region in year *t*, given by

(7) $$T_{wt}=\left(\frac{D_{st}}{D_{t}}\right)T_{st}+\left(\frac{D_{nt}}{D_{t}}\right)T_{nt}$$

In addition, the inter-regional difference contribution $T_{bt}$ refers to the differences of development levels among different regions in year *t*, given by

(8) $$T_{bt}=\left(\frac{D_{st}}{D_{t}}\right)ln\left(\frac{D_{st}/D_{t}}{P_{st}/P_{t}}\right)+\left(\frac{D_{nt}}{D_{t}}\right)ln\left(\frac{D_{nt}/D_{t}}{P_{nt}/P_{t}}\right)$$

According to the above formulas, we can calculate the contribution rate of intra-regional differences $I_{wt}$, the contribution rate of inter-regional differences $I_{bt}$, and the contribution rate of inter-provincial differences of South (North) China $I_{st}$($I_{nt})$ in year t, given by

(9) $$I_{wt}=\frac{T_{wt}}{T_{t}}$$

(10) $$I_{bt}=\frac{T_{bt}}{T_{t}}\;$$

(11) $$I_{st}=\frac{P_{st}}{P_{t}}\frac{T_{st}}{T_{t}}\;$$

(12) $$I_{nt}=\frac{P_{nt}}{P_{t}}\frac{T_{nt}}{T_{t}}$$

### 3.5. Kernel Density Estimation

Kernel density estimation (also known as Parzen window) is a nonparametric method to estimate the probability density function of a random variable [36]. This method does not assume any data distribution and can better reflect the overall distribution of the regional digital economy development level. By observing Kernel density estimation maps in different periods, the dynamic characteristics of the regional distribution can be fully examined.

The density function f(x) of random variable X is estimated as follows [37]

(13) $$f\left(x\right)=\frac{1}{Nh}\overset{N}{\underset{i=1}{\sum }}K(\frac{X_{i}-\bar{x}}{h})$$

(14) $$K\left(x\right)=\frac{1}{\sqrt{2\pi }}\exp\left(-\frac{x^{2}}{2}\right)$$

where K(·) is the kernel function, N is the number of observations, $X_{i}$ is the independent identically distributed observations, $\bar{x}$ is the mean value, and h is the bandwidth. The larger the bandwidth, the smoother the density function curve and the lower the estimation accuracy [38]. In Section 4, we select the data in 2005, 2012 and 2019 as the kernel density estimation map to study the density distribution and dynamic evolution of China’s digital economy development level.

### 3.6. Network Analysis

Groenewold et al. and Li et al. used the VAR model to analyze the spatial spillover effect between regional economies in China [39,40]. We use the VAR model to analyze the dynamic correlation between provinces in digital economy development. The developmental scores of 30 provinces from 2004 to 2019 are used as the original data that are paired to remove the time trend. The premise of the VAR model is a stationary sequence. Thus, the unit root test is carried out on the time series data of each province. If the test results show that the sequence is not stationary, a leveling process (such as difference) is carried out. The VAR model is established for time series data of each two provinces, and the optimal lag order is determined according to the AIC criterion. Based on the Granger causality test, if province A passes Granger causality test to province B, it is considered that the development of the digital economy in Province A has an overflow correlation with province B, if province B passes the test to province A, it is believed that the development of the digital economy in Province A is benefited from province B. According to the correlation, we connect the two provinces by a directed edge. In this way, the spatial network diagram of China’s digital economy development can be obtained.

Network density reflects the correlation degree of digital economic development among provinces. The larger the value, the greater the correlation between provinces. Assuming that there are P provinces, the total number of tested relationships between each two provinces is l, and the maximum number of theoretical relationships is L=P×(P−1). The network density $D_{n}$ is defined as $D_{n}=l/L$.

Centrality measures the importance of each province in the whole network and is generally measured by relative degree centrality and betweenness centrality. If n provinces are directly associated with province A and (P−1) provinces are most likely to be directly connected with province A, the relative degree centrality $D_{r}$ is defined as $D_{r}=n/\left(P-1\right)$.

Betweenness centrality measures the likelihood that one province acts as an ‘intermediary’ between other provinces in the network [41]. Let $G_{BC}$ be the number of shortest paths between provinces B and C. In addition, let $G_{BC}$(A) be the number of shortest paths between provinces B and C and province A, a node in each shortest path. The probability that province A is in the shortest path between province B and C is defined by $Y_{BC}\;\left(A\right)=G_{BC}\left(A\right)$/$G_{BC}$. By adding the probability of all provinces and dividing by the number of pairs of other provinces, we obtain the relative intermediate centrality$\;D_{b}\left(A\right)$, given by

(15) $$\;D_{b}\left(A\right)=\frac{2}{\left(P-1\right)\left(P-2\right)}\;\sum_{B}^{P}\sum_{C}^{P}Y_{BC}\;\left(A\right),A\neq B\neq C,and\;B<C$$

here all *P* provinces are ranked from 1 to *P* for the computation purpose.

## 4. Results

### 4.1. Measurement of Development Levels of Two Regions

**Figure 1:**
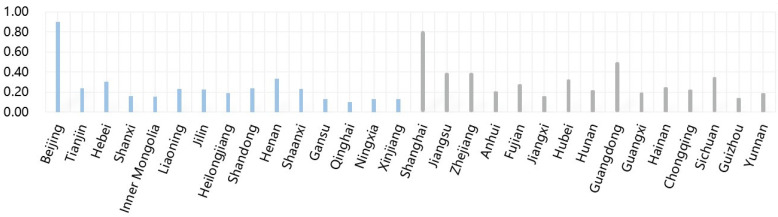
The average scores of the development levels in the 30 provinces.

After obtaining the corresponding data, the development levels of the digital economy in the 30 provinces in China in 2004–2019 are calculated (see Table A2 in the Appendix A). Figure 1 gives the average scores of the development levels in the 30 provinces. The development levels in Beijing and Shanghai are much higher than those in other provinces. In addition, the average scores in 11 southern provinces are greater than 0.2, while only eight northern provinces have such values. Thus, the average development levels of the digital economy in the south are higher than those in the north.

**Figure 2:**
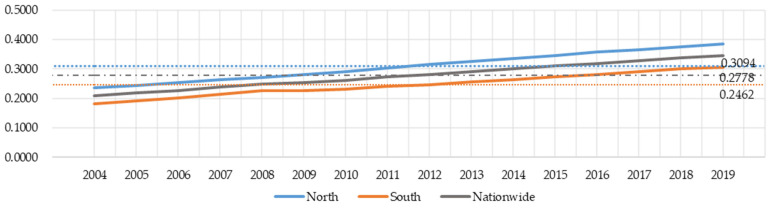
Measurement of development levels of digital economy.

Figure 2 gives the average scores of the development levels in the nationwide, southern, and northern regions from 2004 to 2019. Overall, the average score of the national development level in the past sixteen years is relatively low, which is only 0.2778. However, developmental levels are on the rise, from an average score 0.2086 in 2004 to 0.3465 in 2019. From the regional distributions, the development level in the south is higher than that in the north, and the difference between the southern and northern regions is large. It shows that the development of the digital economy in China is concentrated in the southern region, the development in the northern region is relatively slow.

Table 3 gives the status quo of the development level, which is analyzed by regional classification of the four first-level indicators in Table 1. In terms of digital infrastructure, the southern region has a larger score. The differences between these two regions are striking, indicating that the digital infrastructure construction level in the southern region is higher than that of the northern region.

Regarding the digital innovation ability, the southern region again has a higher score than the northern region. The southern region has strong digital innovation ability due to its advantages in capital investment, scientific research, and higher education. In addition, the southern region has invested more funding in infrastructure, communication technology transformation, and upgrading investment in recent years.

In terms of digital industry scale, the score of the southern region is much higher than that of the northern region. The digital industry chain in the southern region is becoming more complete, and the digital industry has been developing toward large-scale and intensive development. The digital economic output is much higher than that of the northern region. Finally, for digital technology application, the score of the southern region is much higher than that of the northern region, and the northern region is lower than that of the nationwide.

In general, the southern region is ahead of the northern region in all the four aspects, among which the scale of digital industry and the application of digital technology are much more advanced than the northern region.

### 4.2. Results of Theil Index and Subgroup Decomposition

**Figure 3:**
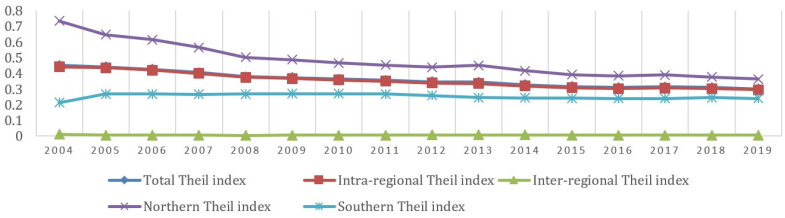
Theil index of China’s digital economy development level.

Figure 3 shows the trend of the Theil index of the development level of Chinese digital economy. From 2004 to 2019, the trend curves of total Theil index and intra-regional Theil index almost coincide, which shows a decreasing trend, indicating that the regional differences of China’s digital economy development mainly come from the intra-regional differences. During the study period, the inter-regional Theil index is between 0.05–0.1, indicating that the inter-regional differences have little influence on the regional difference. The Theil index of the northern region showed a decreasing trend, changing from 0.4525 in 2004 to 0.3469 in 2012 rapidly, and then changing from 0.3440 in 2013 to 0.3011 in 2019 slightly. Obviously, the intra-regional differences mainly come from the inter-provincial differences in the north. The Theil index of the northern region declined rapidly at first and then at a slow rate. The rapid development of China’s Internet from 2004 to the outbreak of mobile Internet in 2012 has largely driven the development level of the digital economy in northern provinces and reduced inter-provincial differences in northern China. The Theil index in the south has not changed much. Therefore, the inter-provincial differences in south China are relatively stable. Some provinces in southern China have obvious geographical advantages, digital industrialization and industrial digitalization have been developing rapidly. For example, the large internet companies Huawei, Alibaba, and Tencent have developed steadily.

**Figure 4:**
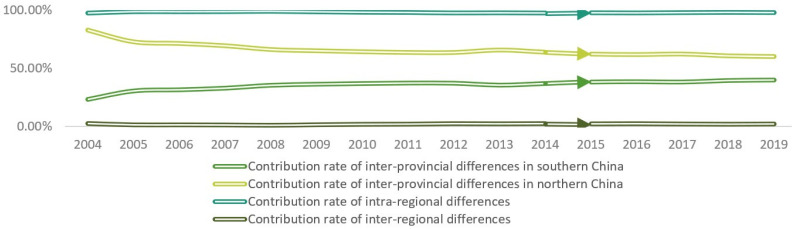
Contribution rates of China’s digital economy development Theil index.

Figure 4 shows that the contribution rates of regional differences in China’s digital economy. The contribution rate of intra-regional differences is the largest one, but the contribution rate of inter-regional differences is the lowest, which is close to 0.00%. From the change trend of the whole period, the contribution rate of inter-provincial differences in the south shows an upward trend, increasing from 23.25% in 2004 to 40.02% in 2019, while that in the north shows a downward trend, decreasing from 82.85% in 2004 to 60.21% in 2019. The four contribution rates show that the differences of the Chinese digital economy mainly come from the inter-provincial differences in the north.

### 4.3. Results of Kernel Density Estimation

**Figure 5:**
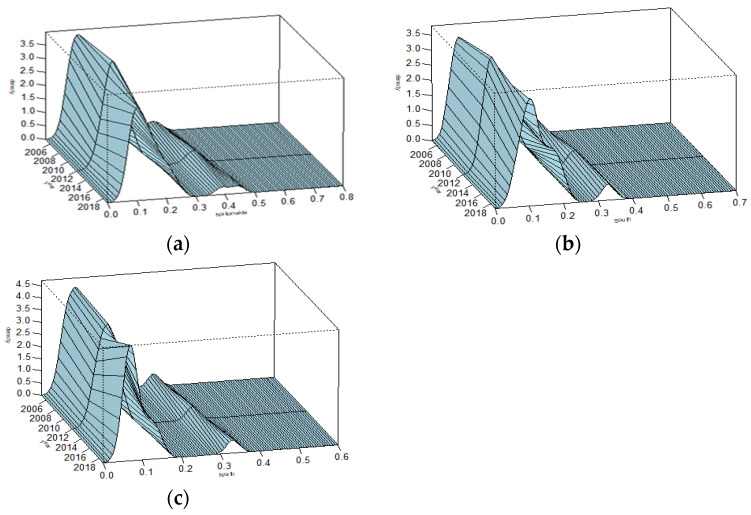
Estimates of kernel density distribution: (**a**) distribution in the whole country; (**b**) distribution in southern China; (**c**) distribution in northern China.

Using 2005, 2012 and 2019 as the test time periods, Figure 5 gives the estimates of density functions for the development levels of the digital economy in the whole country and two regions. In all these three figures, the distributions move to the right while the right tail of the kernel density estimation curve is long, indicating that the development levels of the digital economy in the nation and two regions have been improved in recent years. However, there are still problems regarding the low-level aggregation and uneven spatial distribution. Each of these three kernel density curves have a double peak, which suggests that the development level of digital economy in China is polarizing. The wave peaks in the southern region and whole country have the tendency of becoming shorter and wider, which indicates that the development of the digital economy in China is unbalanced. The southern provinces with high development levels will improve the development level faster, while the provinces with low development levels will improve the development level at a low rate. Therefore, the imbalance of the digital economy development between provinces is becoming more and more obvious.

As time goes on, the peak on the right side of the nationwide kernel density map gradually weakens, which indicates that the distribution of development level in the whole country may shift from a multipolar pattern to a single pattern. In the sample observation period, the wave peaks of the kernel density estimation curve in the northern region first decrease and then increase. The gap between the development levels in different northern provinces first increases and then gradually narrows.

### 4.4. Results of Network Analysis

**Figure 6:**
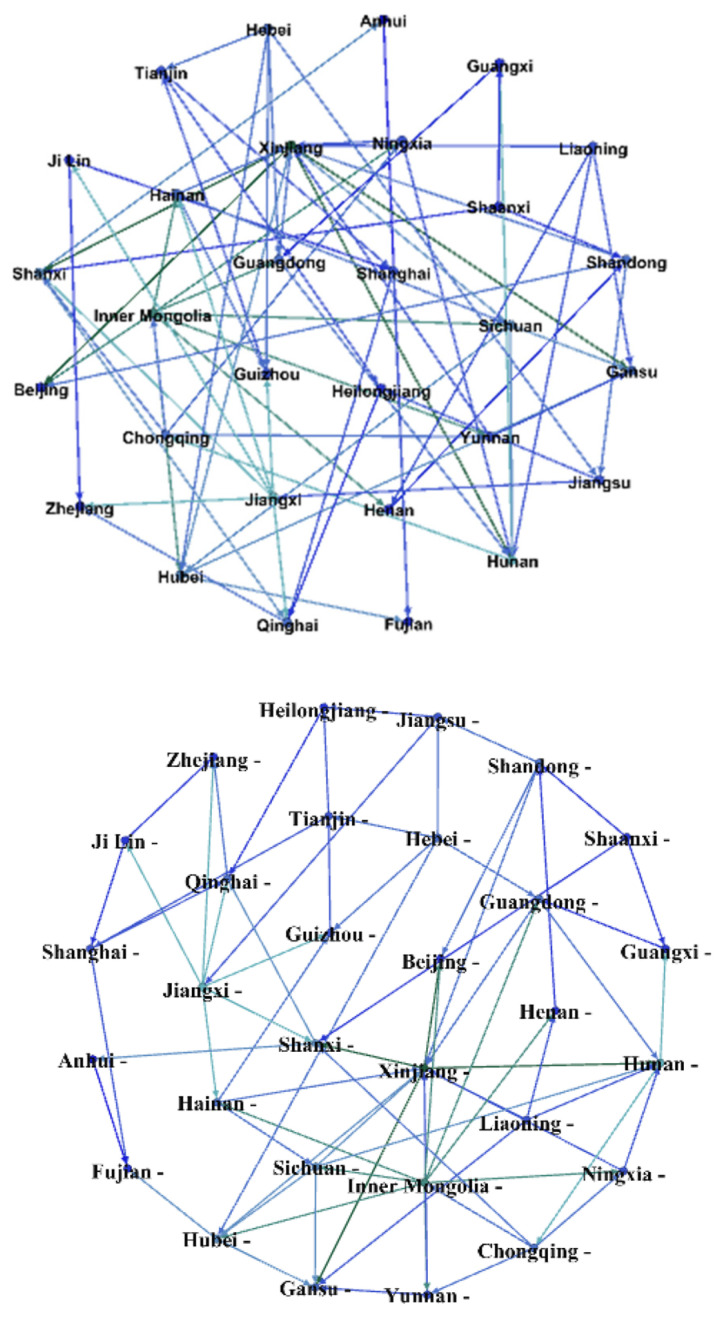
Association map of China’s digital economic development spatial network.

According to the method in Section 3.6, the digital economic development scores of 30 provinces from 2004 to 2019 are tested by the Granger causality test. The results show that 71 associations are identified. Figure 6 gives the association map of China’s digital economic development network using the Gephi software. The spatial correlation is not limited to inter-provincial in the southern region or northern region, but inter-provincial in the whole country. The number of associations within the northern region or the southern region is less than the number of associations between provinces across regions, which is an unexpected result.

Moreover, the spillover effect of association is not restricted by geographical location but exists widely among different provinces. For example, Xinjiang has the largest number of related provinces, and it has spillover effects on the development of the digital economy in Beijing, Gansu, Hunan, and Shanxi. In addition, Guangdong, Hainan, Hubei, Liaoning, Ningxia, Shandong, Sichuan, Yunnan, and Inner Mongolia benefit the development of the digital economy in Xinjiang. Among these provinces, Hunan, Guangdong, Hainan, Hubei, Sichuan, and Yunnan belong to the south, and Xinjiang, Beijing, Gansu, Shanxi, Liaoning, Ningxia, Shandong, and Inner Mongolia belong to the north. Xinjiang is only adjacent to Gansu. Figure 1 shows that the development level of the digital economy in Xinjiang is lower than these provinces except Ningxia. It is expected that Xinjiang should obtain benefits from these provinces with a high level of digital economy development. The question is why Xinjiang has spillover effects on provinces with higher digital economy levels. According to the reports of Xinjiang’s digital economy in recent five years, we find that Xinjiang has built a high-level, technologically advanced digital infrastructure connecting the world. Xinjiang has realized the docking of an optical cable system with neighboring countries such as Kazakhstan and Kyrgyzstan, and thus become an important west-facing inter-national telecommunications network hub in China. This may be the reason why Xinjiang’s digital economy development is relatively low, but it is related to many other provinces in the network.

According to the definition in Section 3.6, the network density of China’s digital economy development is 0.082. This indicates that the inter-provincial correlation is low, and the regional cooperation of digital economy is insufficient. By collaborative development based on regional advantages and provincial advantages, the development speed of digital economy and the development level of China’s digital economy can be accelerated.

Table 4 gives the spatial network centrality of China’s digital economy development. In the south, there are 38 beneficial associations and 33 spillover associations between provinces. On the contrary, there are 33 beneficial associations and 38 spillover associations between provinces in the north. Thus, the south has more benefited effects, but the north has more spillover effects. If the southern provinces with higher levels of digital development can give full play to the spillover effect in the northern provinces in the future, the development level of digital economy in the north will be further improved.

In the south, Jiangxi, Hunan, Hubei, and Sichuan are more associated with other provinces. Their relative degree centralities are higher, which are 0.241, 0.241, 0.207 and 0.207, respectively. In the north, Xinjiang, Inner Mongolia, and Shanxi are more related to other provinces, and their relative degree centralities are 0.414, 0.310, and 0.207, respectively. Among the 30 provinces, the relative degree centralities of Beijing, Shanghai, and Guangdong, which rank top three in digital economy development level, are relatively low. This shows that provinces with a high level of digital economic development have strong spillover potential.

Regarding the betweenness centrality, two southern provinces, Hunan and Chongqing, and two northern provinces, Inner Mongolia and Xinjiang, have values greater than 100. The south and the north are relatively ‘equal’ in terms of the betweenness centrality. Some provinces in both regions play an intermediary role in the spatial network association. The betweenness centrality values of Zhejiang, Fujian, Beijing, Hebei, Liaoning, Shaanxi, and Gansu provinces are 0, which suggests that these provinces do not act as a bridge. This shows that the correlation potential of these provinces needs to be explored.

## 5. Conclusions and Discussion

In recent years, the Chinese digital economy has developed rapidly. However, it is still at a relatively early developmental stage compared with developed countries [42]. The regional economic differences between northern and southern China have strong historical background factors, including economic policies, geographical locations, and resource endowment from the central government. At the same time, the development levels of different provinces are not completely independent. The development of one province often influences other provinces. Using the factor analysis and network analysis methods, this paper analyzes both the developmental differences between the northern and southern regions as well as the spatial correlation relationships between provinces.

Our analysis results show that the development level of the digital economy in China is generally low. The polarized and uncoordinated regional development is significant. In addition, there are benefit-related relationships or spillover-related relationships among some provinces. Specifically, there are mainly the four following aspects. First, the development level of the digital economy in south China is significantly higher than that in the north. Secondly, the regional differences in the development of China’s digital economy mainly come from the inter-provincial differences in the northern region. Third, the disparity between the development of the digital economy in southern provinces tends to widen, while that in the north is likely to narrow. Finally, spatial correlation between provinces is not constrained by region, distance, or development level. Provinces with high development levels may benefit from lower-level provinces.

One of the main reasons for the national low developmental stage is the low level of digital infrastructure construction. Therefore, the key improvement should concentrate on the development of the new generation of information technologies. In particular, the strategies should take advantage of 5G technologies, accelerate the implementation of 5G networks, improve the 5G facilities, and promote the applications of 5G technologies. Meanwhile, we need to accelerate the construction of communication networks and transformation of information in the central and western regions, especially in poor and backward areas, so that information technologies can cover users in a wider range of areas.

The expansion of the digital economy development gap will make more production factors in backward areas flow into developed areas as a cycle, which is not conducive to the realization of common prosperity. Therefore, the future development of the digital economy should focus on the balanced development of the whole country. Backward areas cannot blindly imitate the development model of areas with high development levels of digital economy. Instead, the backward areas should take their comparative advantages as the development engine, such as the geographical location, transportation, and agricultural foundation, to promote the development trend of the digital economy. In addition, the southern region should continue to innovate the development, promote the upgrading of the digital economy industry, and strengthen resource integration with the northern region, drive the development of the northern region, and finally make the national digital economy develop to an internationally high level.

In the follow-up research, we will find methods to quantitatively analyze the benefiial effect and spillover effect between provinces with correlation relationships and identify whether there is causality in the correlation of their digital economy development, especially among provinces with a large developmental level gap. Finally, it is worth noting that, although this work is designed to study the digital economy in China, the proposed research principles, index systems, and statistical computing methods will be applied to study the digital economy in other countries.

## Figures and Tables

**Table 1:** The index system to evaluate the development level of digital economy.

First-Level Indicators	Second-Level Indicators
Digital Infrastructure	Optical density
	Mobile phone base station density
	Port access per square kilometer
	Number of websites per capita
Digital Innovation Capability	Ratio of RD expenditure in GDP of information industry
	The proportion of fixed asset investment in the information industry in the total fixed asset investment of the whole society
	Ratio of technology market turnover to GDP
Digital Industry Scale	The proportion of information industry employees in the employed population
	Software revenue as a percentage of GDP
	E-Commerce sales per capita
	Number of enterprises in information industry
	The proportion of telecommunication business in GDP
Application of digital technology	Mobile phone penetration
	Internet penetration
	Number of 100 people using computers in industrial enterprises
	The number of websites owned by 100 enterprises
	The proportion of enterprises with e-commerce activities

**Table 2:** Two regions in China for analyzing digital economy.

Region	Provinces
Northern region	Beijing, Tianjin, Hebei, Shanxi, Inner Mongolia, Liaoning, Jilin, Heilongjiang, Shandong, Henan, Shaanxi, Gansu, Qinghai, Ningxia, and Xinjiang
Southern region	Shanghai, Jiangsu, Zhejiang, Anhui, Fujian, Jiangxi, Hubei, Hunan, Guangdong, Guangxi, Hainan, Chongqing, Sichuan, Guizhou, and Yunnan

**Table 3:** The index score of the first level indictors.

Level Indicators	Nationwide	South	North
Digital Infrastructure	0.0442	0.0501	0.0411
Digital Innovation Capability	0.0696	0.0759	0.0652
Digital Industry Scale	0.1053	0.1192	0.0971
Application of digital technology	0.0903	0.1000	0.0829

**Table 4:** Spatial network centrality of China’s digital economy development.

Region	Province	Benefit Related	Overflow Related	Related Total	Relative Degree Centrality	Betweenness Centrality
South	Shanghai	2	2	4	0.138	7.250
	Jiangsu	2	2	4	0.138	67.476
	Zhejiang	3	0	3	0.103	0.000
	Anhui	1	1	2	0.069	12.000
	Fujian	3	0	3	0.103	0.000
	Jiangxi	1	6	7	0.241	64.560
	Hubei	3	3	6	0.207	8.810
	Hunan	5	2	7	0.241	185.667
	Guangdong	3	2	5	0.172	40.143
	Guangxi	2	1	3	0.103	16.667
	Hainan	2	3	5	0.172	71.143
	Chongqing	1	4	5	0.172	166.333
	Sichuan	2	4	6	0.207	24.476
	Guizhou	3	1	4	0.138	35.000
	Yunnan	2	2	4	0.138	3.976
North	Beijing	3	0	3	0.103	0.000
	Tianjin	2	2	4	0.138	25.000
	Hebei	0	5	5	0.172	0.000
	Shanxi	4	2	6	0.207	55.524
	Inner Mongolia	1	8	9	0.310	128.667
	Liaoning	0	4	4	0.138	0.000
	Jilin	1	2	3	0.103	7.000
	Heilongjiang	2	1	3	0.103	3.667
	Shandong	2	3	5	0.172	85.810
	Henan	2	1	3	0.103	60.143
	Shaanxi	0	3	3	0.103	0.000
	Gansu	5	0	5	0.172	0.000
	Qinghai	4	1	5	0.172	17.857
	Ningxia	2	2	4	0.138	4.643
	Xinjiang	8	4	12	0.414	138.190

## Data Availability

Not applicable.

## Appendix A

**Table A1:** Data of Beijing for the 17 s-level indictors from 2013 to 2018.

Second-Level Indicators	2013	2014	2015	2016	2017	2018
Optical density	13.09	14.38	16.40	18.25	21.24	22.44
Mobile phone base station density	2869.05	2922.62	3036.90	3113.10	3482.14	3892.86
Port access per square kilometre	706.43	690.42	940.77	1061.90	1082.14	1226.13
Number of websites per capita	2.08	2.12	2.37	2.80	3.25	3.34
Ratio of RD expenditure in GDP of information industry	0.02	0.02	0.02	0.03	0.03	0.03
The proportion of fixed asset investment in the information industry in the total fixed asset investment of the whole society	0.03	0.03	0.03	0.03	0.03	0.05
Ratio of technology market turnover to GDP	0.14	0.15	0.15	0.15	0.16	0.15
The proportion of information industry employees in the employed population	0.05	0.05	0.06	0.06	0.06	0.06
Software revenue as a percentage of GDP	0.21	0.22	0.24	0.25	0.28	0.29
E-Commerce sales per capita	35,307.80	41,879.18	48,505.30	55,346.07	84,687.70	84,778.09
Number of enterprises in information industry	34,519	34,669	31,346	31,523	31,778	31,534
The proportion of telecommunication business in GDP	0.03	0.03	0.04	0.02	0.03	0.05
Mobile phone penetration	159.53	189.46	181.73	178.06	172.85	186.11
Internet penetration	22.71	22.42	22.66	21.90	24.96	29.66
Number of 100 people using computers in industrial enterprises	57	61	62	66	67	70
The number of websites owned by 100 enterprises	59	60	63	64	65	64
The proportion of enterprises with e-commerce activities	7.50	12.60	17.10	18.00	19.00	20.70

**Table A2:** Measurement of the development level of digital economy.

Province	2004	2005	2006	2007	2008	2009	2010	2011	2012	2013	2014	2015	2016	2017	2018	2019
Beijing	0.8149	0.8043	0.8285	0.8522	0.8613	0.8619	0.8505	0.8828	0.8860	0.9476	0.9287	0.9396	0.9508	0.9890	0.9949	0.9956
Tianjin	0.2026	0.2097	0.2153	0.2190	0.2243	0.2240	0.2317	0.2336	0.2359	0.2414	0.2425	0.2482	0.2476	0.2491	0.2605	0.2658
Hebei	0.1820	0.1918	0.2150	0.2395	0.2692	0.2808	0.2937	0.3026	0.3136	0.3290	0.3385	0.3582	0.3602	0.3783	0.3881	0.3915
Shanxi	0.0954	0.0975	0.1100	0.1301	0.1496	0.1461	0.1550	0.1635	0.1722	0.1828	0.1850	0.1862	0.1907	0.1961	0.2103	0.2102
Inner Mongolia	0.0757	0.0981	0.1013	0.1034	0.1347	0.1461	0.1542	0.1598	0.1618	0.1734	0.1792	0.1809	0.1811	0.1923	0.2084	0.2091
Liaoning	0.1874	0.1960	0.2022	0.2183	0.2203	0.2206	0.2215	0.2286	0.2376	0.2383	0.2427	0.2457	0.2579	0.2609	0.2699	0.2047
Jilin	0.1853	0.1954	0.1988	0.2081	0.2153	0.2153	0.2297	0.2335	0.2301	0.2322	0.2364	0.2375	0.2384	0.2395	0.2403	0.2411
Heilongjiang	0.1355	0.1394	0.1651	0.1714	0.1831	0.1834	0.1948	0.1926	0.1973	0.2020	0.2135	0.1905	0.2145	0.2147	0.2145	0.2190
Shandong	0.1691	0.1723	0.1719	0.1760	0.1818	0.1817	0.1972	0.2199	0.2337	0.2564	0.2799	0.2818	0.2923	0.3136	0.3375	0.3569
Henan	0.2430	0.2585	0.2685	0.2762	0.2872	0.2910	0.3075	0.3200	0.3281	0.3394	0.3693	0.3921	0.3995	0.4120	0.4138	0.4319
Shaanxi	0.1604	0.1960	0.1940	0.2154	0.2181	0.2187	0.2201	0.2293	0.2309	0.2353	0.2528	0.2538	0.2579	0.2646	0.2675	0.3111
Gansu	0.0831	0.0912	0.0988	0.0957	0.1096	0.1096	0.1184	0.1296	0.1227	0.1340	0.1399	0.1453	0.1573	0.1679	0.1826	0.2197
Qinghai	0.0184	0.0380	0.0443	0.0479	0.0797	0.0797	0.0516	0.0623	0.0720	0.0665	0.0898	0.1794	0.1940	0.1883	0.2266	0.2001
Ningxia	0.1129	0.1234	0.1282	0.1287	0.1307	0.1277	0.1273	0.1277	0.1292	0.1294	0.1281	0.1240	0.1315	0.1385	0.1474	0.1551
Xinjiang	0.0600	0.0758	0.0811	0.1170	0.1280	0.1281	0.1294	0.1389	0.1391	0.1423	0.1462	0.1516	0.1516	0.1527	0.1673	0.1911
Shanghai	0.7122	0.7231	0.7264	0.7810	0.7858	0.8018	0.8075	0.8106	0.8292	0.8454	0.8491	0.8550	0.8554	0.8626	0.8700	0.8758
Jiangsu	0.3052	0.3163	0.3469	0.3586	0.3631	0.3729	0.3734	0.3828	0.3866	0.3995	0.4166	0.4411	0.4479	0.4583	0.4624	0.4681
Zhejiang	0.3110	0.3196	0.3354	0.3496	0.3517	0.3615	0.3654	0.3762	0.3892	0.4032	0.4111	0.4305	0.4589	0.4715	0.4732	0.4993
Anhui	0.1192	0.1258	0.1365	0.1432	0.1485	0.1582	0.1653	0.1691	0.2022	0.2454	0.2578	0.2605	0.2779	0.2847	0.2945	0.3151
Fujian	0.2195	0.2274	0.2122	0.2233	0.2291	0.2499	0.2526	0.2621	0.2826	0.3077	0.3179	0.3287	0.3299	0.3385	0.3421	0.3510
Jiangxi	0.0764	0.0780	0.0853	0.0835	0.0918	0.0805	0.0909	0.1474	0.1777	0.1986	0.2117	0.2266	0.2306	0.2371	0.2437	0.2541
Hubei	0.2385	0.2417	0.2683	0.2812	0.3118	0.3281	0.3352	0.3412	0.3299	0.3355	0.3460	0.3554	0.3669	0.3719	0.3869	0.3962
Hunan	0.1348	0.1350	0.1450	0.1492	0.1524	0.1694	0.1983	0.2270	0.2370	0.2419	0.2592	0.2695	0.2799	0.2808	0.2953	0.3027
Guangdong	0.4558	0.4650	0.4621	0.4708	0.4779	0.4825	0.4913	0.4960	0.5037	0.5127	0.5246	0.5265	0.5362	0.5386	0.5412	0.5445
Guangxi	0.1075	0.1275	0.1385	0.1486	0.1596	0.1696	0.1798	0.1897	0.1926	0.2030	0.2170	0.2122	0.2421	0.2612	0.2981	0.3128
Hainan	0.2184	0.2261	0.2349	0.2363	0.2382	0.2418	0.2428	0.2438	0.2478	0.2504	0.2526	0.2632	0.2642	0.2707	0.2760	0.2845
Chongqing	0.1489	0.1641	0.1551	0.1546	0.1799	0.1805	0.1901	0.2117	0.2320	0.2269	0.2485	0.2585	0.2822	0.2994	0.3216	0.3220
Sichuan	0.2572	0.2731	0.2813	0.2859	0.2934	0.3207	0.3386	0.3465	0.3592	0.3659	0.3791	0.3812	0.3999	0.4094	0.4398	0.4429
Guizhou	0.0738	0.0818	0.1098	0.1152	0.1184	0.1286	0.1386	0.1494	0.1510	0.1511	0.1539	0.1630	0.1738	0.1795	0.1870	0.2011
Yunnan	0.1543	0.1578	0.1609	0.1646	0.1754	0.1763	0.1805	0.1955	0.1989	0.2019	0.2050	0.2070	0.2108	0.2133	0.2155	0.2219
